# Chemoselective decarboxylation of ceiba oil to diesel-range alkanes over a red mud based catalyst under H_2_-free conditions†

**DOI:** 10.1039/d2ra00853j

**Published:** 2022-06-08

**Authors:** Nur Athirah Adzahar, N. Asikin-Mijan, Mohd Izham Saiman, G. Abdulkareem Alsultan, M. S. Mastuli, Mohd Razali Shamsuddin, Y. H. Taufiq-Yap

**Affiliations:** Catalysis Science and Technology Research Centre, Faculty of Science, Universiti Putra Malaysia UPM Serdang Selangor 43400 Malaysia taufiq@upm.edu.my; Department of Chemistry, Faculty of Science, Universiti Putra Malaysia UPM Serdang Selangor 43400 Malaysia mohdizham@upm.edu.my +603-9769 7238; Department of Chemical Sciences, Faculty of Science and Technology, Universiti Kebangsaan Malaysia UKM Bangi 43600 Selangor Darul Ehsan Malaysia ckin_mijan@yahoo.com nurul.asikin@ukm.edu.my; School of Chemistry and Environment, Faculty of Applied Sciences, Universiti Teknologi MARA Shah Alam Selangor 40450 Malaysia; Preparatory Centre for Science and Technology, Universiti Malaysia Sabah Kota Kinabalu Sabah 88400 Malaysia; Faculty of Science and Natural Resources, Universiti Malaysia Sabah Kota Kinabalu Sabah 88400 Malaysia taufiqyap@ums.edu.my

## Abstract

Concerns over global greenhouse gas emissions such as CO_*x*_ and NO_*x*_ as well as the depletion of petroleum fossil resources have motivated humankind to seek an alternative energy source known as green diesel. In this study, green diesel was produced *via* a deoxygenation (DO) reaction of ceiba oil under a H_2_-free atmosphere over Ni modified red mud-based catalysts, which have been synthesized *via* a precipitation – deep-deposition assisted autoclave method. The obtained catalyst was further characterized by XRF, XRD, BET, FTIR, TPD-NH_3_, FESEM, and TGA. Based on the catalytic activity test, all Ni/RMO_*x*_ catalysts facilitated greater DO activity by yielding 83–86% hydrocarbon yield and 70–85% saturated diesel *n*-(C_15_ + C_17_) selectivity. Ni/RMO_3_ was the best catalyst for deoxygenizing the ceiba oil owing to the existence of a high acidic strength (12717.3 μmol g^−1^) and synergistic interaction between Fe–O and Ni–O species, thereby producing the highest hydrocarbon yield (86%) and *n*-(C_15_ + C_17_) selectivity (85%). According to the reusability study, the Ni/RMO_3_ could be reused for up to six consecutive runs with hydrocarbon yields ranging from 53% to 83% and *n*-(C_15_ + C_17_) selectivity ranging from 62% to 83%.

## Abstract 2

Concerns over global greenhouse gas emissions such as CO_*x*_ and NO_*x*_ as well as the depletion of petroleum fossil resources have motivated humankind to seek an alternative energy source known as green diesel.

## Graphical abstract

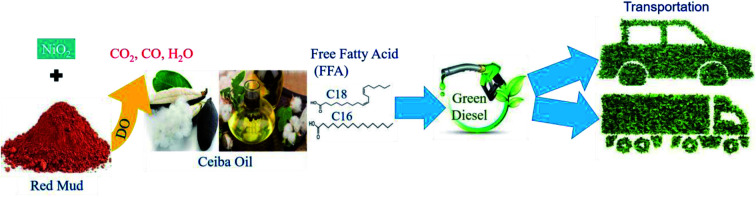

## 1. Introduction

The global warming issue and depletion of fossil fuels are the two most threatening problems of our present day civilization. According to NASA,^1^ in 2021, CO_2_ levels have indeed reached 416 ppm. This recent inexorable rise in CO_2_ levels reveals a highly consistent link with the use of fossil fuels. The resulting rise in CO_2_ levels will continue to melt the ice in Antarctica, resulting in a ‘doomsday glacier’, causing global sea levels to rise and affecting people all over the world. Aside from that, the global energy crises are the outcome of the potential future depletion of fossil fuel. Renewable sources of energy such as green diesel are generally considered a replaceable alternative to traditional fossil fuels due to their similar physicochemical properties criteria, feedstock availability, and environmental friendliness.^2–4^ In general, green diesel can be produced *via* catalytic hydrodeoxygenation (HDO) and deoxygenation (DO) of oxygenated compounds (triglycerides and fatty acid devatives). In HDO processes, oxygenated species are removed under high pressure conditions with the presence of H_2_, whereas in DO processes, oxygenated species are removed in the form of CO, CO_2_, and H_2_O *via* decarbonylation/decarboxylation (deCO_*x*_) pathways under H_2_-free conditions.^5,6^ Due to the fact that no H_2_ is needed in the DO process, DO pathways are usually preferred.

Ceiba pentandra, often known as kapok or silk-cotton tree, is a tropical tree of the Malvaceae family that grows quickly (up to 13 feet per year).^7^ Pentandra is a drought-tolerant tree and grown in waste land thus readily and abundantly available.^8^ The pods of these trees are leathery, ellipsoid, pendulous capsules that are 10–25 cm long and 3–6 cm in diameter. The capsules split open into five valves, revealing a mass of woolly, yellowish grey, and glossy fibre in which 120–175 seeds are lodged.^9^ Ceiba seeds are blackish in colour and have a composition of 13% water, 5% ash, 20% crude fibre, 6% fat, 29% protein, and 20% carbohydrates. Apparently, ceiba seeds consist of a yellow and pleasant oil that is within the range of 20–28% by weight with high amount of free fatty acid (FFA) (FFA = 6%).^3^ As a result, ceiba oil is an inedible feedstock that has been used successfully for oil sorption and biodiesel generation utilising a variety of catalysts.^10–12^ Ceiba oil has not been used for green diesel generation, hence it was employed as a prospective feedstock source in this study. Apparently, ceiba seeds consist of a yellow and pleasant oil that is within the range of 20–28% by weight with high amount of free fatty acid (FFA) (FFA = 6%).^3^ As a result, ceiba oil is an inedible feedstock that has been used successfully for oil sorption and biodiesel generation utilising a variety of catalysts.^10–12^ At present, the transformation of ceiba oil to diesel is rarely reported in DO studies. Thus, in the present study, special focus will be focused on the DO of ceiba oil to green diesel.

A catalyst is a substance that increases the rate of a chemical reaction without being consumed in the process. Many studies in the literature have reported on the DO process using noble metal catalysts such as Pd, Pt, and Ru.^13–16^ However, noble metal catalysts are expensive, which limits it applicability for commercialization. As a result, the utilisation of industrial waste or naturally occurring solids containing catalytically active metals such as Fe, Ni, V, and others as a substitute for commercial catalysts can assist in minimizing the cost of catalyst utilization.^17,18^ Bauxite residue (BR), often known as red mud, is made during alkali leaching of bauxite and is considered a low-grade Fe ore containing 30% to 60% Fe and Al (10–20%).^17,19,20^ Because of its high alkalinity, currently, its storage sites pose a significant safety and environmental risk.^21^ It is noteworthy to mention that red mud waste disposal is an expensive operation that accounts for 5% of the production cost.^22^ Indeed, red mud is mainly composed of Fe (30–60%), which can be considered an ideal precursor for synthesizing an effective DO catalyst.^23^ Fe has recently been discovered to have strong oxophilic effects and favours a robust redox reaction, making it easier to break C–C and C–O bonds.^24,25^ Likewise, a red mud catalyst has demonstrated an excellent plastic pyrolytic activity, producing ∼67% liquid fuel yield.^26,27^ Previous reports also affirmed that the presence of Fe species in red mud resulted in a good organic liquid yield (17–74%) but noted that those findings were limited to bio-oil production.^28–30^ Several studies on gasification production processes have shown that a Fe-catalyzed system can prevent coking activity and prolong the life span of the catalyst. Apparently, strong basic sites of Fe will transform the coke by assisting the Boudouard reaction, in which carbon combines with CO_2_ to produce CO gas.^31^ Up to now, the Fe species is also a good metal support, as it is capable of improving the surface area of the catalyst and permits homogenous dispersion of the active metal, thus effectively rendering excellent deoxygenation activity.^32,33^ Indeed, the use of a modified red mud catalyst for green diesel production *via* DO has not yet been investigated. As a result, the current research will focus on the creation of a red mud-based catalyst modified with Ni for the production of green diesel from DO of ceiba oil.

## 2. Experimental

### 2.1 Materials and chemicals

Red mud was collected from three different red mud waste areas; Semabok, Bachang, and Durian Tunggal from Melaka. *Ceiba Pentandra* L. seeds were purchased from West Java, Indonesia. The ceiba oils were extracted from the seeds by using a cold-pressing method.^34^ These feedstocks were used for the DO reaction without further treatment and purification. The physicochemical properties of the feedstock were determined using the American Oil Chemists' Society (AOCS) method, and the results are tabulated in Table 1.^35^ The majority of the ceiba oil consists of palmitic acid (C16:0) at 19.2%, oleic acid (C18:1) at 17.4%, linoleic acid (C18:2) at 39.6%, and malvaloyl*18 CE (18:CE) at 18.5%. Nickel(ii) nitrate hexahydrate (Ni(NO_3_)_2_·6H_2_O, purity >99%) was purchased from R&M, Malaysia. Concentrated hydrochloric acid (HCl, 85–87% purity) was purchased from J. T. Baker (USA). Ammonia solution (NH_3_, 30% purity) was purchased from R & M Chemical (UK). Solvents such as ethanol, hexane, and acetone were acquired by Merck & Co., USA. Analytical grade *n*-hexane (purity >98%, Merck, Malaysia) and absolute ethanol (purity >98%) were used as solvents. A standard solution that consists of alkanes and alkenes (C_8_–C_20_) and an internal standard 1-bromohexane (CHBR, purity >98% (GC grade)) for gas chromatography (GC) analysis were purchased from Sigma Aldrich, Malaysia and used without further purification. *n*-Hexane (GC grade) with a purity >98% from Merck (Germany) was used for dilution (2.1%).

**Table 1:** The physiochemical properties of ceiba oil^a^

Oil properties	Ceiba oil	Method
Acid value (mg_KOH_ g^−1^)	11.9	AOCS Ca 5a-40
FFA value (%)	5.9	AOCS Ca 5a-40
Fatty acid composition of oil (%)		AOCS Cel-62 & Cel-661
Palmitic (C16:0)	19.2	
Oleic (C18:1)	17.4	
Linoleic (C18:2)	39.6	
Malvaloyl (18:CE)	18.5	

a Traces% of lauric acid and myristic acid (0.1%), palmitoleic acid ‘(0.3%), stearic acid (2.6%), linolenic acid (1.5%), arachinic acid (0.56%) and others (0.34%).

### 2.2 Preparation of the Ni supported red mud catalyst

Approximately 30 g of three different types of red muds (RM_*x*_, *x* = 1, 2, 3) were separately ground and dissolved in mixtures of 150 mL of 37% HCl and 100 mL of distilled water. The mixtures were then stirred for 24 h at 30 rpm and centrifuged. Subsequently, the obtained liquid was further titrated with 30% ammonia that had been diluted 1 : 1 with distilled water until the liquid reached a pH of 11. The precipitant was then filtered, washed with distilled water, and dried in the oven for 24 h. The obtained solid was then calcined at 550 °C for 3 h under atmospheric pressure, and all the catalysts were denoted as RMO_*x*_: RMO_1_, RMO_2_, and RMO_3_. The deep-deposition technique was used in order to dope the Ni promoter on the RMO_1_, RMO_2_, and RMO_3_. Ni was successfully been deposited by slowly adding a 0.6 M nickel salt solution into RMO_1_. The Ni deposition was performed by adding 1 M ammonia dropwise until the pH reached 10 under vigorous stirring. The slurry was then put into an autoclave machine at 220 °C for 24 h. The obtained solid was then filtered and washed using distilled water. The product was dried in the oven overnight and calcined at 550 °C for 3 h. The catalyst was denoted as Ni/RMO_1_. Similar steps were repeated for RMO_2_ and RMO_3_. The catalysts were denoted as Ni/RMO_2_ and Ni/RMO_3_. These catalysts were further reduced under a H_2_ atmosphere at 550 °C for 3 h, and the catalysts were denoted as Ni/rRM_1_, Ni/rRM_2_, and Ni/rRM_3_.

### 2.3 Catalyst characterization

The identification of the crystallography and structural properties of all catalysts was carried out using powder X-ray diffraction (XRD). The XRD analyses were performed using a Shimadzu diffractometer, model XRD-6000, with a scan speed of 4 °C min^−1^ with a 2*θ* range within 5° to 40°. The diffractograms that were produced were matched with the published International Centre for Diffraction Data (ICDD) files in order to determine the crystallinity phases of the synthesized materials. The Thermo-Finnigan Shopmatic 1990 series N_2_ sorption analyser was used to analyse the specific surface area and pore distribution using N_2_-adsorption and desorption techniques. The catalysts were degassed for 12 h at 150 °C to eliminate contaminations and moisture on the catalyst surface. The adsorption and desorption processes of N_2_ were evaluated in a vacuum chamber at −196 °C. The acidity of the catalysts were investigated using temperature-programmed desorption with NH_3_ as the probe molecules (TPD-NH_3_). The analysis was performed using a Thermo-Finnigan TPDRO analyzer model 1100 equipped with a corresponding thermal conductivity detector (TCD). Approximately 0.05 g of sample was added to a quartz tube, and the sample was initially pre-treated at 150 °C under N_2_ conditions to remove excess moisture. The pre-treated samples were exposed to NH_3_ for 1 h at room temperature for NH_3_ adsorption. Subsequently, the excess NH_3_ was flushed out using N_2_ (20 mL min^−1^) at room temperature for 35 min. The treated catalyst was heated from 50 °C to 900 °C at a heating rate of 10 °C min^−1^ in a flow of He (30 mL min^−1^). The desorbed NH_3_ was detected by the TCD. The acidic sites was calculated by using eqn (1):

Acidic sites,1
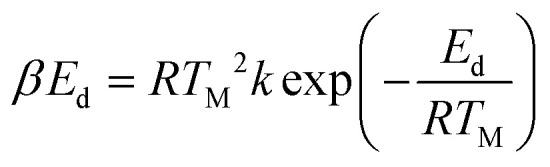
where *β* is the strength of the binding energy, *E*_d_ is the activation desorption energy, *T*_M_ is the maximum temperature of TPD spectrum, *R* is the ideal gas constant (8.314 kJ per mol per kelvin), and *T* is absolute temperature. The surface morphology and elemental composition of the catalysts were observed using a field emission scanning electron microscope and energy dispersive X-ray (FESEM/EDX) (Hitachi S-3400N). The elemental analysis was also estimated using X-ray fluorescence spectroscopy (XRF) (Rigaku, RIX 3100). The XRF was operated at 50 kV and 70 mA using a wavelength dispersive spectrometer that was equipped with a rhodium tube, LiF 200 crystal, and scintillation counter. The chemical functional group of the comprised catalyst was determined using a PerkinElmer (PC) Spectrum 100 FTIR with a resolution of 4 cm^−1^ within the range of 300–4000 cm^−1^. A thermogravimetric analysis, TGA instrument (TGA 1000i, Instrument Specialists Inc., USA) was used to determine the thermal stability of the catalyst under an inert environment. The powder sample was first placed in an alumina crucible and then heated from 25 °C to 900 °C at a heating rate of 30 °C min^−1^ under nitrogen gas flow rate of 40 mL min^−1^.

### 2.4 DO reaction of ceiba oil

**Fig. 1:**
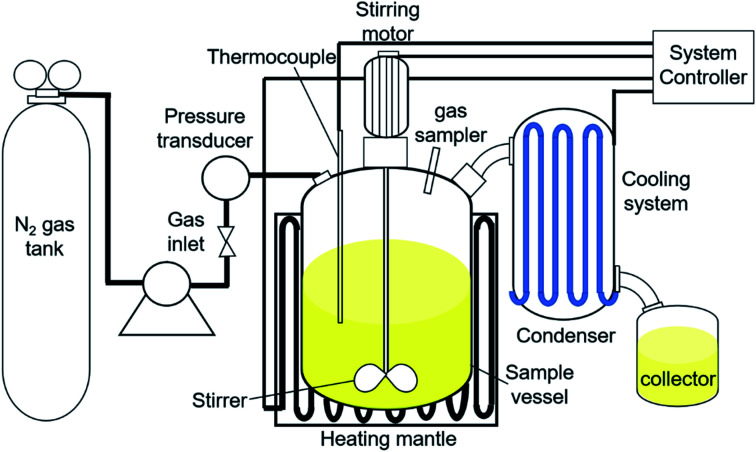
Reactor set-up for DO of ceiba oil.

The catalytic DO reaction of ceiba oil was performed in a 250 mL mechanically stirred semi-batch reactor under inert N_2_ flow (Fig. 1). Basically, the DO reaction of ceiba oil was carried out at 350 °C under the flow of inert gases (50 cc min^−1^) for 2 h using 10 g of ceiba oil and 3 wt% catalyst loading. Prior to the reaction, the oxygen in the reactor was removed by purging with N_2_ gas at a flow rate of 20 cc min^−1^. The inert N_2_ gas was then continuously flowed at this rate. Vapor or volatile species generated during the DO process was condensed into the liquid product using an external water cooling circulator at 22 °C and collected using a vessel collector. The liquid product was then evaluated by using the gas chromatography-flame ionisation detector (GC-FID) and gas chromatography-mass spectrometer (GC-MS); meanwhile, the gaseous product was collected using a sampling gas bag and further analysed by using gas chromatography-thermal conductivity detector (GC-TCD). A thermogravimetric analysis, TGA instrument (TGA 1000i, Instrument Specialists Inc, USA) was used to determine the extent of coke formation on the spent catalyst in an oxidative environment. The powder sample was first placed in an alumina crucible and then heated from 25 °C to 900 °C at a heating rate of 30 °C min^−1^ under oxygen flow rate of 40 mL min^−1^.

### 2.5 Product characterization

The liquid products were identified using alkane and alkene standards (C_8_–C_20_) procured from Sigma-Aldrich (USA). The liquid product was analyzed qualitatively using GC-FID (Shimadzu GC-14B) equipped with an HP-5 capillary column (length of 30 m; inner diameter of 0.32 mm; film at 300 °C) based on the previous study by Baharudin *et al.*^36^ First, the liquid product was diluted with GC grade *n*-hexane. 1-Bromohexane was used as an internal standard for quantitative analysis. A 1 mL aliquot of a sample was injected into the GC column. The injection temperature was 250 °C, and nitrogen gas served as the carrier gas. The initial temperature of the oven was set at 40 °C, and it was held there for 6 min; then, it was ramped up to 270 °C at a heating rate of 7 °C min^−1^.^37^ Literature data also supported the GC-FID oven programme method.^38–40^ The green diesel conversion, product selectivity, and hydrocarbon yield were defined as in eqn (2) and (3).^41^2
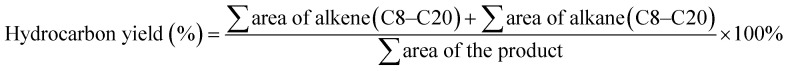
3
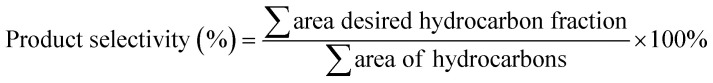


The organic compounds in the liquid product were further investigated by a GC–MS (model Shimadzu QP2010 Plus) fitted with a Zebron ZB-5 MS column (30 m × 0.25 mm × 0.25 μm) using a splitless inlet. The liquid deoxygenated products were diluted to 100 ppm using GC-grade hexane (purity >98%). The component peaks in the GC-MS spectrum were identified from the National Institute of Standards and Testing (NIST) library based on a probability of agreement of ≥95%.^42^ The selectivity of the deoxygenated products was determined by using eqn (4):4
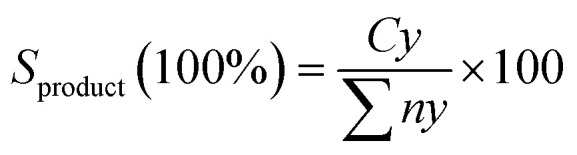
where *S*_product_ is the yield of the organic compound (%), *Cy* is the area of the desired organic compound, and ∑*ny* is the total area of the organic compounds. The chemical functional group of a comprised liquid product was determined using a PerkinElmer (PC) Spectrum 100 FTIR with a resolution of 4 cm^−1^ in the range of 300–4000 cm^−1^. To quantify the concentration of the gases (CO_2_, CO)^43^ obtained from the DO reaction, 1 μL of gas was injected into an off-line gas chromatograph (GC) (Agilent G1540N, USA) connected to a thermal conductivity detector (TCD).

## 3. Results and discussion

### 3.1 Characterization of fresh red mud

**Fig. 2:**
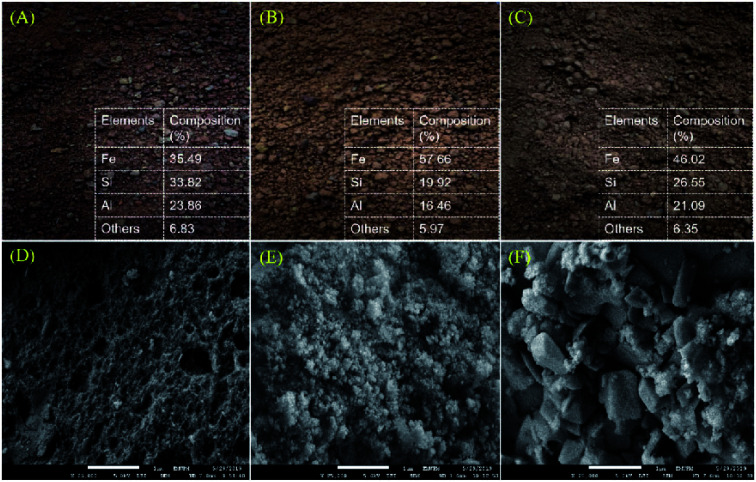
Picture and EDX result for (A) RM_1_, (B) RM_2_ (C) RM_3_ and FESEM images of (D) RM_1_, (E) RM_2_ (F) RM_3_. *others: * = TiO_2_, FeO(OH), Al(OH)_3_.

TGA analysis was performed on fresh red mud 1–3 (RM_*x*_), and the results are displayed in Fig. S1.† Based on the TGA analysis, minor weight loss (3–8%) was observed in the temperatures range of ∼50–200 °C owing to the elimination of physically absorbed water, Al_2_O_3_·3H_2_O (Al_2_O_3_·3H_2_O → Al_2_O_3_ + 3H_2_O) and chemically bounded water, 2 (Al(OH)_3_ → Al_2_O_3_ + 3H_2_O) on the RM_*x*_ particles.^44,45^ This chemically bound water might come from the dehydration of the hydrate molecule in the minerals and the decomposition of gibbsite phases.^46^ Notably, detection of rich Fe species in all fresh red mud was confirmed by FESEM-EDX analysis. The Fe content in RM_2_ was the highest (58%), while others were 35–46% (Fig. 2A–C). The Fe% in RM_*x*_ was in accordance with previous findings (30–60%).^47–49^ It is noteworthy to mention that all the RM_*x*_ were red in colour, which is due to the rich Fe(iii) oxide species that comprise approximately 20–60% of its mass.^50,51^ The morphology structure of RM_*x*_ is displayed in Fig. 2D–F. There are obvious variations in the surface morphology. Fig. 2D–F shows the morphology structure of the red mud. It is clear that RM_1_ exhibits a spongy-like structure. Meanwhile, RM_2_ shows an assembly of small aggregates, and RM_3_ exhibits agglomerated particles. Indeed, when compared to RM_1_ and RM_3_, the Fe rich red mud (RM_2_) exhibits a greater homogeneity of the aggregate size; this reveals that the high Fe content in red mud contributes to the homogeneity of the aggregate size of the material.^52–54^ Fig. S2† shows the XRD patterns for RM_*x*_. The majority of the RM_*x*_ displayed peaks belonging to hematite (Fe_2_O_3_, ICDD card no. 00-006-0502), aluminium oxide hydrate (Al_2_O_3_·3H_2_O, ICDD card no. 00-001-0307), quartz (SiO_2_, ICDD card no. 00-001-0649), and anatase (TiO_2_, ICDD card no. 00-001-1292).^55,56^ Similarly, insignificant peaks were also discovered for other elements such as goethite (FeO(OH)) and gibbsite (Al(OH)_3_).^57^ This finding was found to be in accordance with the weight loss of chemically bounded water molecule in the findings of the TGA analysis.

### 3.2 Characterization of the red mud based nickel catalyst

As explained in Section 2.2, the RM_*x*_ was further calcined at 550 °C for 3 h to fully transform the RM_*x*_ to the RMO_*x*_ species-based catalyst, which was further characterized by XRF analysis. The Fe content (referred to as Fe_2_O_3_ species) in all RMO_*x*_ species was found to be within the range of 97–99% (Table 2). Because RMO_*x*_ is primarily composed of Fe species, it has a great potential to be used as a catalyst. Further Ni inclusion (20 wt%) resulted in a significant reduction in the Fe content (53–74%), whereas the Ni species (referred to as NiO_2_ and NiO) were discovered to be in the range of 26–38%. The Ni species were prominent in Ni/RMO_1_ (38%), suggesting that the rich porous structure of RMO_1_ (Fig. 2D) has the ability to trap more Ni species into the pores during the catalyst synthesis.^58^ Notably, the Ni and Fe contents were reduced slightly (∼2–5%), indicating that a few of the oxide species (Fe_2_O_3_, NiO_2_, NiO) in the reduced catalysts successfully converted to metallic Fe and Ni.

**Table 2:** Crystallite size and elemental composition of the RMO_*x*_, Ni/RMO_*x*_ and Ni/rRM_*x*_ catalysts

Catalyst	Crystallite size (Fe_2_O_3_) 2*θ*: 33.4°	Elemental composition (%)		
		Fe_2_O_3_	NiO_2_/NiO	Others^a^
RMO_1_	172.4	99.1	—	0.9
RMO_2_	129.4	97.0	—	3.0
RMO_3_	129.4	97.1	—	2.9
Ni/RMO_1_	64.7	61.2	37.2	1.7
Ni/RMO_2_	64.7	64.8	34.6	0.6
Ni/RMO_3_	64.7	73.0	26.3	0.7
Ni/rRM_1_	96.3	60.2	38.0	1.8
Ni/rRM_2_	64.7	67.2	32.5	0.2
Ni/rRM_3_	96.3	70.5	28.7	0.8

a Other = SiO_2_, TiO_2_, Al(OH)_3_.

**Fig. 3:**
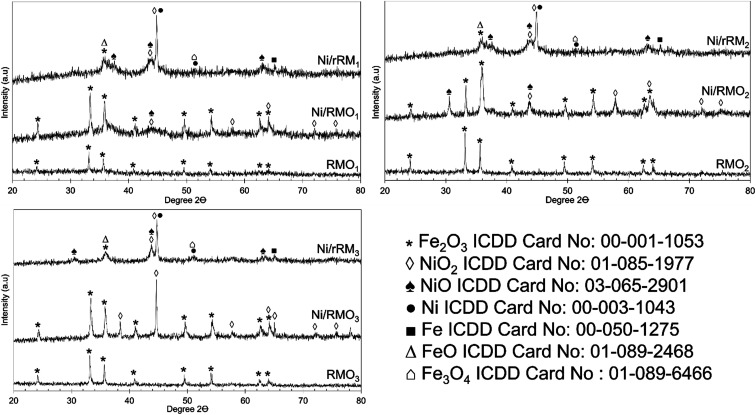
XRD diffractogram of RMO_*x*_, Ni/RMO_*x*_ and Ni/rRM_*x*_ catalysts.

The XRD patterns of the RMO_*x*_, Ni/RMO_*x*_ and Ni/rRM_*x*_ catalysts are shown in Fig. 3. The XRD of RMO_*x*_ exhibited a hematite structure of Fe_2_O_3_ at 2*θ* = 24.3°, 33.4°, 35.8°, 38.4°, 49.7°, 54.3°, and 62.7° (ICDD card no. 00-001-1053) on the RMO_*x*_, Ni/RMO_*x*_, and Ni/rRM_*x*_. The NiO_2_ (ICDD card no. 01-085-1977) and NiO (ICDD card no. 03-065-2901) species were detected on all Ni/RMO_*x*_ catalysts. As for Ni/rRM_*x*_, typical peaks corresponding to NiO, metallic Ni, Fe_2_O_3_, Fe_3_O_4_, FeO, and metallic Fe were observed. The formation of metallic Ni and Fe indicated the successful H_2_ gas reduction of Ni^2+^ to Ni^0^ and Fe^3+^ to Fe^0^. The existence of the oxide species after the H_2_ reduction method suggested that at low temperatures, the H_2_ method (550 °C for 3 h) does not completely reduce the nickel oxide and iron oxides. Indeed, previous studies concurred that reducing nickel oxide to metallic nickel requires temperatures ranging from 600 to 900 °C for 1–3 h,^59–61^ whereas reducing hematite to metallic iron takes 4–10 h at temperatures ranging from 650 to 1000 °C. Altogether, all red mud catalysts dominantly owing to alkaline oxide species (Fe_2_O_3_), indicating that all these catalysts are very resistant to coking and particularly boost the catalyst lifespan.

**Fig. 4:**
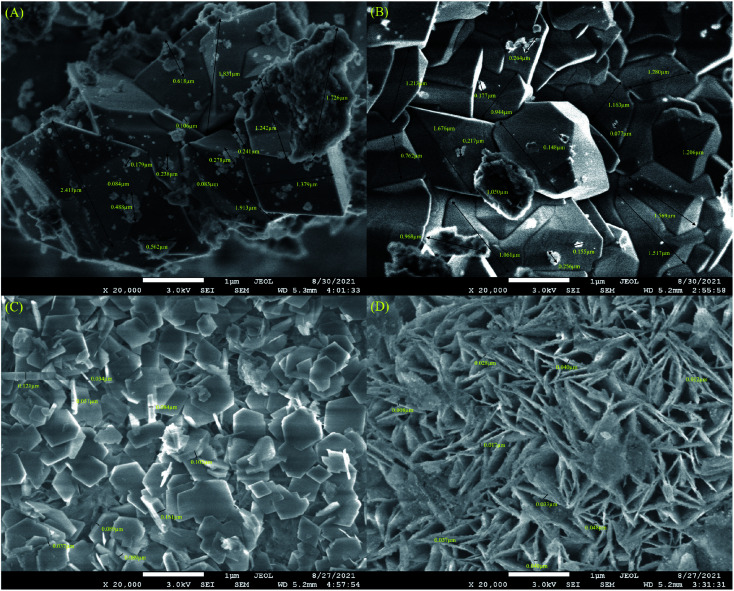
FESEM images of (A) RMO_1_, (B) RMO_3_, (C) Ni/RMO_3_ and (D) Ni/rRM_3_ catalysts.

The surface area, total pore volume, and mean pore diameter of the synthesized catalysts were determined using the BET and BJH methods, and the results are tabulated in Table 3. Among the RMO_*x*_ catalysts, RMO_1_ had the highest surface area (135 m^2^ g^−1^) compared to RMO_2_ and RMO_3_. Upon the addition of Ni, the surface area of the Ni/RMO_*x*_ catalysts gradually decreased, implying the successful deposition of the Ni species on the RMO_*x*_ surface.^62^ In contrast, for Ni/RMO_3_, the surface area increased remarkably after the addition of the Ni species. The reduction of the surface area is ascribed to the coverage of surfaces and the blockage of pores by the Ni species. Fig. S3B† affirmed this finding, whereby Ni/RMO_3_ displayed a type IV H2 isotherm with a large hysteresis gap, while Ni/RMO_1_ and Ni/RMO_2_ catalysts demonstrated a Type III H3 isotherm with a narrow hysteresis gap, illustrating a narrow range of pore necks due to pore blockage (Fig. S3A–C†).^63^ The surface area of the reduced catalysts was lower than that of the RMO_*x*_ and Ni/RMO_*x*_ counterparts. This discovery, which is also consistent with prior research, demonstrates that reducing red mud catalysts to temperatures >400 °C reduces the surface area of the metal oxide-based catalysts.^64^ Notably, there is no link between pore size and pore volume trends with the lowering of the surface area on Ni/rRM_*x*_. As demonstrated in Fig. S4A,† RMO_*x*_ catalysts had mesopores with pore diameters varying from 2 to 50 nm. Meanwhile, in Ni/RMO_*x*_ and Ni/rRM_*x*_ catalysts, multiscale pores (meso- and macro-pores) with pore sizes ranging from 2 to 50 nm and 50 to 130 nm were discovered, with Ni/rRM_2_ showing the highest pore volume and diameter (Fig. 4B and C).^43^

**Table 3:** Textural properties of RMO_*x*_, Ni/RMO_*x*_ and Ni/rRM_*x*_ catalysts

Catalysts	N_2_ adsorption–desorption analysis		
	Surface area^a^ (m^2^ g^−1^)	Pore size diameter^b^ (nm)	Pore volume^b^ (cm^3^ g^−1^)
RMO_1_	135	7.8	0.27
RMO_2_	77	9.0	0.18
RMO_3_	73	7.4	0.14
Ni/RMO_1_	108	15.0	0.27
Ni/RMO_2_	58	13.3	0.19
Ni/RMO_3_	83	9.5	0.20
Ni/rRM_1_	97	9.3	0.23
Ni/rRM_2_	50	53.8	0.68
Ni/rRM_3_	56	15.4	0.21

a Measured by BET analysis.

b Measured by BJH analysis.

The acid density and strength of acidity for the RMO_*x*_ and modified RMO_*x*_ catalysts were measured using TPD-NH_3_. According to Abdulkareem *et al.* (2016),^65^ the acid site plays a critical role in controlling the C–O cleavage activity of the feedstock toward the desired hydrocarbon product, and this study claimed that the DO activity preferred a catalyst with rich weak and medium acidic sites, as it enables the oxygenated species to be removed from the fatty acid derivatives *via* deCO_*x*_ pathways. The results showed that all the catalysts exhibited weak (*T*_max_ <250 °C), medium (*T*_max_ >250 °C < 500 °C), and strong (*T*_max_ >500 °C) (Table 4 and Fig. S5A–C†) acidic sites, whereby strong acidic sites predominated.^66^ Despite this fact, the strong acidity of the catalyst was desirable to provide the catalytic C–C cracking, leading to the formation of light hydrocarbons (alkanes). The trend in the acidity profiles of catalysts is as follows: Ni/rRM_3_ > Ni/rRM_2_ > Ni/rRM_1_ > Ni/RMO_3_ > Ni/RMO_1_ > Ni/RMO_2_ > RMO_1_ > RMO_2_ > RMO_3_. Ni/rRM_3_ has the strongest acidic sites, while RMO_3_ has the weakest acidic sites. The result also showed that only RMO_3_ has weak and medium sites. This result confirms the feasibility of distributing the Ni species for tuning and maximizing the acidity, hence simultaneously shifting the weak and medium acid sites to strong acid sites. A similar finding was observed in a previous study;^67^ hence, it can be concluded that Ni species play an important role in designing a strong acidic catalyst. Further thermal reduction treatment results in the majority of the Ni/rRM_*x*_ catalysts exhibiting a remarkable increase in the strong acid sites. It is believed that removal of the oxide species on Ni/RMO_*x*_ effectively exposes the Ni^0^ species and Fe^0^, which in turn enhances its acidity. The finding is consistent with those of prior works showing the significant role of Ni^0^ acidic compounds at the SBA-15 and Al_2_O_3_ support surfaces.^68^ A similar observation was found on Fe^0^-rich catalysts.^64^ Because Ni/rRM_3_ exhibited a significant amount of acidic sites (40799.6 μmol g^−1^), the morphological analysis for RMO_3_, Ni/RMO_3_, and Ni/rRM_3_ was performed. The result was also compared with the highest surface area catalysts (RMO_1_). It is worth noting that the largest RMO_1_ particles (particle size: 0.0083–0.6 μm, 1.2–2.4 μm) (Fig. 4A) had no correlation with the formation of the large surface area (Table 3). RMO_3_ has larger particles that were coated with small aggregates (particle size: 0.0077–0.2 μm, 1.0–1.7 μm) (Fig. 4B). Yet, these aggregates went unnoticed with the addition of Ni (Fig. 4C), implying that the increase in the surface area of the Ni/RMO_3_ catalyst is related to the homogeneous particle RMO_3_ distribution by Ni. In this study, indeed, the addition of the Ni species and the H_2_ reduction approach resulted in significant changes in the morphology of the RMO_3_ support. The FESEM micrograph shows that Ni/RMO_3_ has plate-like structures (0.037–0.16 μm thick), while the FESEM micrograph of Ni/rRM_3_ shows that it forms uniform nanosheets (0.008–0.048 μm thick) decorated by Ni foam (Fig. 4D). This implies that the iron and nickel oxides (see XRD) must have been largely consumed during the H_2_ reduction approach. The resulting uniform nanosheets of Ni/rRM_3_ are possibly interesting for applications in DO reactions because of the presence of both meso- and macropores.

**Table 4:** Acidity profiles of RMO_*x*_, Ni/RMO_*x*_ and Ni/rRM_*x*_ catalysts

Catalysts	Acidity strength^a^ (μmol g^−1^)		
	Weak <200 °C	Medium >200 °C < 500 °C	Strong >500 °C
RMO_1_	—	2955.5	2955.5
RMO_2_	—	2955.5	2621.4
RMO_3_	205.9	503.3	877.8
Ni/RMO_1_	—	—	10445.9
Ni/RMO_2_	—	—	5975.9
Ni/RMO_3_	—	—	12717.3
Ni/rRM_1_	—	25 274.7	19042.2
Ni/rRM_2_	—	—	25381.7
Ni/rRM_3_	—	—	40799.6

a Determined by TPD-NH_3_.

**Fig. 5:**
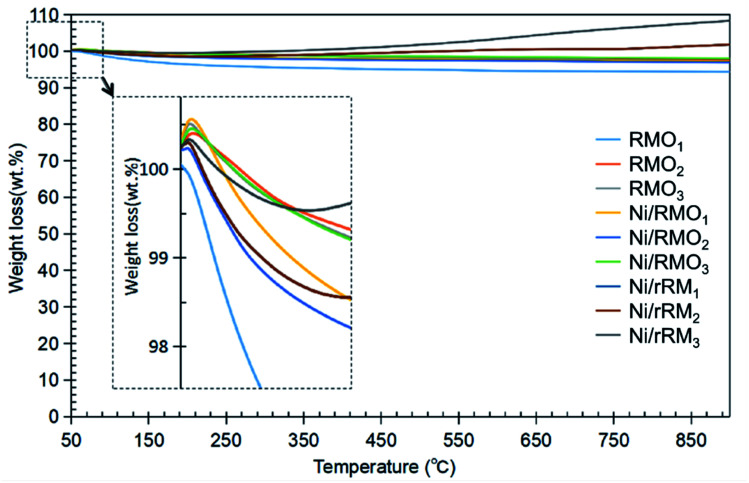
TGA thermogram for RMO_*x*_, Ni/RMO_*x*_ and Ni/rRM_*x*_ catalysts.

TGA analysis was performed to investigate the stability of the catalysts, and the result is shown in Fig. 5. All of the catalysts had insignificant weight loss during thermal treatment up to 850 °C, suggesting that all catalysts have good stability. The addition of Ni and the H_2_ reduction approach barely changed the stability of the red-mud based catalyst, implying that the red-mud based support catalyst itself preserves its stability. When compared with the findings of the acidity trend (see Table 3), the increase in the acid sites does not play a critical role in improving the stability of the modified red mud-based catalyst. Our result is unlike Kaya's finding,^69^ who demonstrated that acid modification of red mud followed by calcination apparently increased the thermal stability of the red-mud based material. To summarize, the correlation between stability and acidity should be further investigated. Based on the TGA result, all the catalysts exhibited two stages of weight gain (∼1–4%) at temperatures from 50–200 °C and 450–850 °C (3–5%) due to the adsorption of the water and N_2_ adsorption.^70^ The weight gained at 450–850 °C was dominated by the Ni/rRM3 catalyst, which was attributed to the formation of multi-scale (meso- and macro-) porosity structures (Fig. S4C†) that increased the tendency of N_2_ adsorption, generating Ni_3_N, Fe_2_N, and ε-Fe_3_N 71–74. This finding is aligned with previous studies showing that N_2_ can form a bond with the metal to form a nitride compound at high temperatures (>400 °C).^71–75^

### 3.3 Catalytic DO profile of ceiba oil

**Fig. 6:**
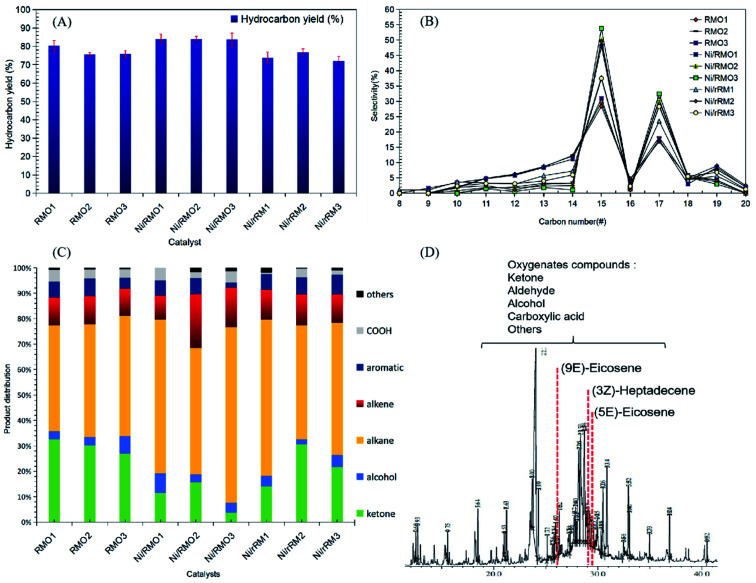
Data collected from catalysed DO liquid product (A) hydrocarbon yield and (B) *n*-(C_15_ + C_17_) selectivity, (C) product distribution and (D) GCMS of deoxygenated compound. Reaction condition: *T* = 350 °C, 2 h reaction time, 3 wt% of catalyst loading.

From Table 1, the fatty acid composition of ceiba oil is mainly composed of 39.6% linoleic acid (C18:2), 17.4% oleic acid (C18:1), 18.5% malvaloyl*18 CE (C18 : CE), and 19.2% palmitic acid (C16:0). The C_16_ and C_18_ fatty acid derivatives will undergo the deCO_*x*_ reaction to form *n*-heptadecenes (*n*-C_17_) and *n*-pentadecenes (*n*-C_15_).^76^ A blank experiment was performed under the same condition to determine the product distribution during the DO of ceiba oil without the presence of the catalyst. The blank reaction exhibited a low *n*-(C_8_–C_20_) hydrocarbon yield (<20%) and low *n*-C_15_ + C_17_ selectivity, implying that a catalyst was required to produce a higher yield and selectivity of the deoxygenated product (Fig. 6A and B). In the case of the catalysed reaction, the majority of the reaction shows an excellent catalytic DO reaction performance. Ni/RMO_3_ yielded the highest hydrocarbon fraction (∼86%), while Ni/rRM_3_ showed the poorest DO activity with a hydrocarbon yield of ∼73%. This could be due to the presence of a high proportion of strong acidic sites in Ni/rRM_3_ (40799.6 μmol g^−1^, see Table 4), which prone toward C–C cracking than C–O cleavage activity. Furthermore, the large pore size of Ni/rRM_*x*_ catalysts (see Table 3) also allowed molecules to diffuse across acid active areas, resulting in further cracking and the formation of volatile species.^77,78^ In the case of Ni/RMO_3_, the preferential amount of strong acidic sites (12717.3 μmol g^−1^) enables them to have a high DO activity, which is likely to undergo C–O bond cleavage. Indeed, the Ni/RMO_*x*_ catalysed process promotes outstanding DO activity and *n*-C_15_ + C_17_ selectivity compared to RMO_*x*_ and Ni/rRM_*x*_. This finding strongly affirmed that the interaction between the Ni–O species and Fe–O phase motivated the deCO_*x*_ activity. The deterioration of the DO activity by Ni/rRM_*x*_ is due to the generation of rich O-vacancies (Ni^0^, Fe^0^) that can be confirmed through XRD results showing existence of metallic Ni and Fe peaks in all Ni/rRM_*x*_ catalysts (see Fig. 3). These rich-O vacancies species that act as acid sites (see TPD-NH_3_, the acidity of Ni/rRM_*x*_ > Ni/RMO_*x*_) will invoke the occurrence of extensive undesired C–C cracking and result in coking, which reduces the DO activity.^64,68^ This finding is aligned with prior studies showing that rich metallic Ni (Ni^0^) supported on Al_2_O_3_ and SBA-15 catalysts is easily deactivated during the DO reaction due to the high coking activity (∼17–60%).^68^ Fig. S6† shows the number of saturated *n*-C_15_ + C_17_ is significantly higher than the number of unsaturated *n*-C_15_ + C_17_, suggesting that metal sites were the reason for the greater hydrogenation activity.^79,80^ The hydrogenation reaction may occur as a result of the H_2_ produced *in situ* through the cracking (C–C cleavage) reaction of the deoxygenated product.^81^ In addition, saturation of the deCO_*x*_ product can be achieved *via* the occurence of decarboxylation. It is noteworthy to mention that the high strength acidic sites (Table 4) favoured the formation of a high degree of saturated hydrocarbon species *via* the decarboxylation reaction. Overall, the occurrence of the decarboxylation and hydrogenation reactions indicates the existence of a rich saturated hydrocarbon species.

The DO activity of all catalysed DO reactions was monitored by FTIR analysis, and the result is shown in Fig. S7.† The FTIR spectra of ceiba oil showed the main absorption bands at 2917 cm^−1^ and 2850 cm^−1^ (–CH stretching), 1740 cm^−1^ (–C=O ester stretching), and 1120 cm^−1^ (C–O–C stretching). Based on an earlier work,^82^ Putra *et al.* proposed that the DO activity can be evaluated by comparing the decrease in the C=O and C–O–C peak intensities. This is confirmed, as there is a slight shift of the C=O peak at 1740 cm^−1^ in ceiba oil to 1702 cm^−1^ in the liquid products, indicating transformation of carboxylic acid from ester *via* triglyceride cracking. The following results are also in agreement with the GC-MS results, whereby the oxygenated species were discovered to be less in all DO products, while aliphatic alkanes and alkenes were the principal components that were detected (Fig. 6C).^83^ Notably, the DO reaction of ceiba oil catalyzed by Ni/RMO_3_ showed the highest amount of alkane hydrocarbon fractions within the range *n*-(C_8_–C_20_) with product distribution 70%; meanwhile, the lowest alkane hydrocarbon fraction is 42% for RMO_1_. Again, this shows that the Ni/RMO_3_ catalyst favors the formation of saturated hydrocarbon-like fuel.

According to the results, the amount of hydrocarbon detected by GC-FID and GC–MS did not complement very well. This is primarily due to the ability of Fe-promoted catalysts to facilitate isomerization reactions that yield hydrocarbon isomer compounds (*Z*) and (*E*), such as 5,9-eicosene (*E*) and 3-heptadecene (*Z*) that have been detected through the GC–MS analysis (Fig. 6D).^84^ Despite the fact that both GC-FID and GC–MS analysis yielded slightly different hydrocarbon yields in the *n*-(C_8_–C_20_) range, it is obvious that straight chain hydrocarbons (saturated and unsaturated) are the major compound in the deoxygenated liquid product. Based on the GC-MS finding, the deoxygenated liquid product exhibited the presence of oxygenated species (ketones, alcohols, carboxylic acids), and the oxygenated species was the highest (47%) for the RMO_1_ catalysed DO reaction due to the high alkalinity of red mud.^85^

### 3.4 Mass balance

A mass balance profile for the DO reaction of ceiba oil into the liquid hydrocarbon product using RM_*x*_, Ni/RMO_*x*_, and Ni/rRM_*x*_ is tabulated in Table 5. According to eqn (4), theoretically, the DO reaction of ceiba oil will produce the hydrocarbon liquid product *via* deCO_*x*_ by releasing CO_2_, CO, and H_2_O as by-products. Hence, the mass fractions including the ceiba oil feedstock, liquid product, and by-products (CO_2_ gas, CO gas, water) will be recorded. Based on the results, a low mass fraction of the liquid product (<12 wt%) was obtained experimentally compared to the theoretical value (68 wt%) with a deviation of <55 wt%. These contradictory results were most readily explained by the formation of undesirable by-products (char + residue), which amounted to a total of 41–46 wt% remaining in the semi-batch reactor after the reaction. Vitolo *et al.* reported that the formation of the by-products (char + residue) was caused by a low degree of volatilization of ceiba during DO at a high temperature (350 °C).^86^ The majority of the Ni-containing catalysts resulted in a high mass fraction of the liquid product (∼11 wt%), especially Ni/RMO_3_. This might be due to the preferential amount of strong acidic sites (12717.3 μmol g^−1^), contributing to a good balance of the occurrence of C–O cleavage and limiting secondary cracking that leads to the formation of volatile species.^3^ In contrast, Ni/rRM_3_ has the lowest mass fraction of the liquid product (4.73 wt%), which might be due to the high acidity site (see TPD-NH_3_) (40799.6 μmol g^−1^), that prone to have an aggressive catalytic cracking. This also could be confirmed, as Ni/rRM_3_ produces the highest fraction of gaseous (48.37 wt%) as the by-product compared to other catalysts. Besides, it is worth noting that all the catalysed DO reactions produced a small quantity of water (3 wt%), which may be produced by fatty acid hydrolysis or the decarbonylation process, but this amount was insignificant because it readily evaporated into gas during the high temperature of the DO reaction. Overall, Ni/RMO_3_ has proven to be effective in deoxygenizing the ceiba oil due to the formation of more condensable liquid-fuel products.

**Table 5:** Mass balance profile for catalytic DO of ceiba oil

Theoretical deCO_*x*_	Ceiba → liquid (oil) + 3 mol CO_2_/CO (g) + 3 mol H_2_O (aq) + by product (4)								
Reaction^a^	Feedstock	Liq-product^b^		Gas^c^		Water^d^		Char + residue^e^	
	(g)	(g)	(wt%)	(g)	(wt%)	(g)	(wt%)	(g)	(wt%)
Theoritical data (deCO_*x*_)	10.00	6.89	68.90	2.49	24.90	0.62	6.20	—	—
RMO_1_	10.12	0.64	6.32	5.10	50.00	0.04	0.40	4.38	43.28
RMO_2_	10.08	0.71	7.04	5.21	51.39	0.04	0.40	4.15	41.17
RMO_3_	10.04	0.94	9.36	4.79	47.70	0.09	0.90	4.22	42.03
Ni/RMO_1_	10.07	1.08	10.64	4.50	44.69	0.10	1.00	4.39	43.59
Ni/RMO_2_	10.01	1.02	10.19	4.48	43.86	0.08	0.80	4.52	45.15
Ni/RMO_3_	10.02	1.09	10.88	4.67	46.61	0.04	0.39	4.22	42.12
Ni/rRM_1_	10.05	1.02	10.15	4.30	43.48	0.12	1.19	4.54	45.17
Ni/rRM_2_	10.05	1.02	10.15	4.30	42.69	0.20	2.00	4.54	45.17
Ni/rRM_3_	10.15	0.48	4.73	5.74	48.37	0.20	1.97	4.56	44.93

a Deoxygenation condition: reaction temperature of 350 °C, 60 min reaction time, 3 wt% of catalyst, under inert condition with 400 rpm stirring rate.

b Mass fraction for Liq-product = [(mass of liq-product/mass of feedstock) × 100].

c Material fraction for gas = [(mass of feedstock − mass of liq-product − mass of (char + residue) − mass of water)/mass of feedstock × 100].

d Material fraction for water = [(mass of water/mass of feedstock) × 100].

e Material fraction for (char + residue) (*Y*) = [(mass of (char + residue)/mass of feedstock) × 100].

### 3.5 Optimization studies

**Fig. 7:**
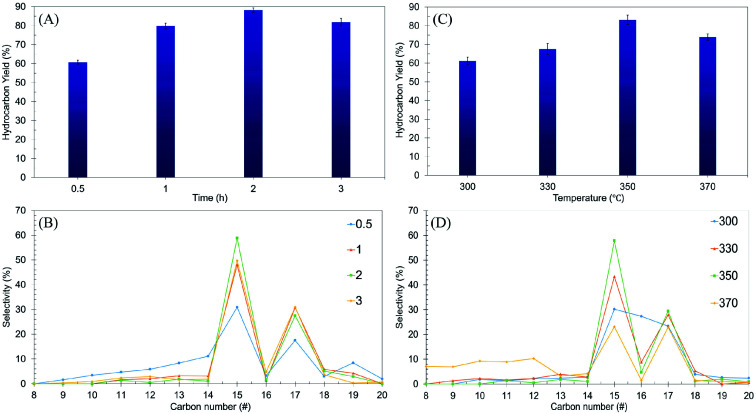
Optimization studies of ceiba: (A and B) the effects of reaction time on hydrocarbon yield and *n*-(C_15_ + C_17_) selectivity at reaction parameters: 350 °C and 3 wt% catalyst loading, (C and D) the effects of reaction temperature on hydrocarbon yield and *n*-(C_15_ + C_17_) selectivity at reaction parameters: 3 wt% catalyst loading and 2 h reaction time with a stirring rate of 300 rpm and under an inert atmosphere.

Using the one-variable-at-a-time technique, the effects of reaction time (0.5–3 h) and reaction temperature (300–370 °C) were examined using the Ni/RMO_3_ catalyst. The impact of reaction time on hydrocarbon yield and *n*-(C_15_ + C_17_) selectivity was investigated and reported in a volcano shape graph (Fig. 7A and B). The lowest hydrocarbon yield (60%) and *n*-(C_15_ + C_17_) selectivity (42%) were achieved when the reaction time was within 0.5 h. This demonstrated that for 0.5 h, there is a low degree of DO activity due to an insufficient amount of energy for the catalyst to initiate the reaction.^5^ Also, when the time was prolonged up to 2 h, the hydrocarbon yield and *n*-(C_15_ + C_17_) selectivity increased remarkably, suggesting that a longer residence time was required for a high degree of interaction between the reactant molecule and the catalyst surface.^87^ Beyond 3 h of reaction time, the DO activity decreased. The hydrocarbon yield and *n*-(C_15_ + C_17_) selectivity decreased from 89% to 81% and 86% to 79%, respectively. Notably, the light hydrocarbon fractions increased from 2% to 7% due to the cracking reaction at longer reaction times.^88^ According to the findings, the most efficient period for the DO reaction is 2 h, which results in a hydrocarbon yield of 89% and *n*-(C_15_ + C_17_) selectivity of 86%.

The effect of reaction temperature was further studied, and the result is shown in Fig. 7C and D. The results showed that increasing the temperature from 300 °C to 350 °C resulted in a significant increase in the hydrocarbon yield (from 61% to 83%) and *n*-(C_15_ + C_17_) selectivity (from 53% to 87%). Further increase of the reaction temperature to >350 °C resulted in a decrease in the DO activity. The *n*-(C_15_ + C_17_) selectivity was significantly reduced, indicating that thermal cracking is favored at high temperatures, resulting in the formation of volatile species and light fractions.^89^ This finding is in agreement with an increase in the *n*-(C_8_–C_12_) selectivity from 3% to 42%. Overall, it can be inferred that reaction time and reaction temperature have a substantial influence on the DO of ceiba oil. Therefore, the optimum conditions for the DO reaction are the use of a 3 wt% catalyst loading for a 2 h reaction time at 350 °C under N_2_ flow.

The composition of the gases collected from the Ni/RMO_3_ catalysed DO reaction process at optimum reaction conditions was further analysed by GC-TCD, and the result is shown in Fig. S8.† Theoretically, the DO of ceiba oil in the absence of H_2_ will favour the decarboxylation and decarbonylation reactions and produce CO_2_ and CO.^90^ However, only CO_2_ was detected by GC-TCD, confirming that the oxygenate species were removed *via* decarboxylation pathways. Aside from that, H_2_ gas was detected, which could be prompted by the cracking process and the water–gas-shift reaction (WGS). The WGS reaction is reversible, and the equation is as follows: CO + H_2_O → CO_2_ + H_2_. The CO gas remained undetected. The absence of the CO gas in the TCD analysis is due to the effective WGS reaction.^91^

### 3.6 Reusability and stability of the Ni/RMO_3_ catalysts

**Fig. 8:**
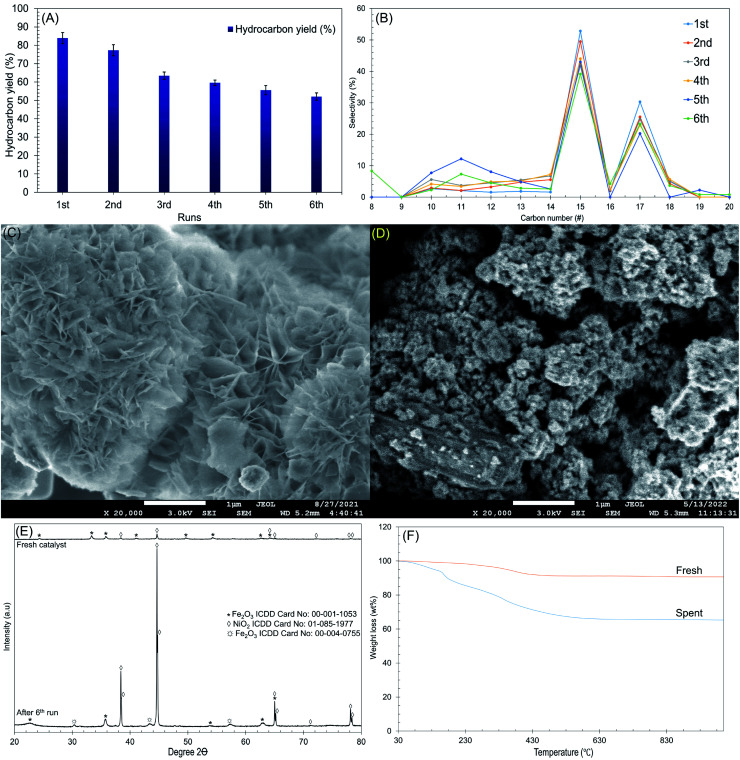
Data collected from the Ni/RMO_3_ catalyst's deoxygenated liquid product after the 6th run at *T* = 350 °C, 2 h reaction time, 3 wt% of catalyst loading (A) hydrocarbon yield, (B) *n*-(C_15_ + C_17_) selectivity with number of DO cycles, (C) FESEM images for the fresh catalyst, (D) FESEM images for the spent catalyst, (E) TGA of Ni/RMO_3_ catalyst, and (F) XRD diffractogram of the catalyst.

The reusability and stability of Ni/RMO_3_ were further investigated at the following reaction condition: 2 h reaction, 350 °C reaction temperature using 3 wt% catalyst loading under N_2_ flow. The result is shown in Fig. 8A and F. To remove adsorbed organics, the catalyst from each cycle was washed numerous times with hexane. The reusability was studied for six cycles, and the gradual reduction in the hydrocarbon yield and *n*-C_15_ + C_17_ was observed (Fig. 8A and B). After the 6th run, the structure of the catalyst transformed from plate-like to agglomeration, indicating that the metal oxides has been sintered (Fig. 8C and D). Apparently, sintering of metal oxides largely occurs by high temperature reaction processes and results in the loss of active sites, thereby reducing the DO activity.^92^ Furthermore, XRD results revealed that the intensity of the NiO_2_ peak at *2θ*: 38.4°, 65.1° and 78.2° (ICDD card no. 01-085-1977) were dramatically increased on the spent catalyst. This is because the NiO_2_ in the fresh catalyst exhibited as an amorphous form has been oxidised throughout the reaction and transitioned to crystalline form, resulting into distinct intensity in the NiO_2_ peak of the spent catalyst. Noted, high DO reaction also lead to structural transformation, which was proven by the new formation of *syn*-maghemite cubic Fe_2_O_3_ which appeared at *2θ*: 30.3°, 44.7° and 57.4° (ICDD card no. 00-004-0755) on spent catalyst (Fig. 8E).^93^ Considering the existence of rhombohedral hematite Fe_2_O_3_ in a fresh catalyst, high thermal reaction temperatures apparently lead to structure distortion and alteration.^94^ Apart from that, TGA analysis also confirmed that the employed catalysts suffered from coke coverage (Fig. 8F). According to the TGA results, the oxidation of the deposits from these reactions occurred at a comparatively higher decomposition temperature (550 °C), with coke deposits accounting for 23 wt%, as suggested by the presence of hard coke. Soft coke (decomposition temperature = 160 °C) was also found but in smaller quantities (7 wt%). Based on this finding, it can be suggested that the coke deposits on Ni/RMO_3_ are mainly polynuclear aromatic coke. Overall, Ni/RMO_3_ after DO suffer with loss of actives sites by sintering and coking but the coke content was lower (46–60 wt%) than prior study.^37,68^ It should be noted that Ni/RMO_3_ is made from waste bauxite, which has been catastrophic in taking lives and devastating surrounding areas. As a result, this research is critical for developing an alternative strategy on how waste management can be used to provide long-term value, such as alternative sources of metal recovery with low cost.^95^

## 4. Conclusion

The potential of red-mud derived catalysts (RMO_*x*_, Ni/RM*O*_*x*_, and Ni/rRM_*x*_) for the chemoselective reaction on the DO of ceiba oil was successfully investigated. The results show that the removal of oxygenated species in the production of diesel-range alkanes *n*-(C_15_ + C_17_). Based on the DO profile, the liquid hydrocarbon yield increased in the order of Ni/RMO_3_ > Ni/RMO_1_ > Ni/RMO_2_ > RMO_1_ > Ni/rRM_2_ > RMO_3_ > RMO_2_ > Ni/rRM_1_ > Ni/rRM_3_. The Ni/RMO_3_ was found to have an effective DO activity with a hydrocarbon yield of ∼86% and *n*-(C_15_ + C_17_) selectivity of up to 85%. This was attributed to a synergistic impact between Ni–O and Fe–O species in the catalyst as well as high-strength acidic sites, which enhance the C–O cleavage, thereby promoting the DO activity. It was also shown that Ni/RMO_*x*_ catalysts enhance the formation of saturated alkanes with selectivity ranging from 49% to 70%, owing to the high strong acidic active sites that promote the saturation process, resulting in the production of more alkanes. Based on the optimization study, reaction time and reaction temperature play a critical role in improving the DO activity. A high hydrocarbon yield (83%) and *n*-(C_15_ + C_17_) selectivity (87%) were observed under the optimum conditions: 3 wt% of catalyst loading, 2 h of reaction time at 350 °C reaction temperature. Despite the fact that the Ni/RMO_3_ catalyst showed tremendous potential for converting ceiba oil to diesel fuel, it was obscured by coke, particularly hard coke, which produces aromatic species (ketones, alcohols, and carboxylic acids) that deactivate the catalyst. To summarise, transformation of waste bauxite to an effective DO catalyst demonstrated an efficient management of waste bauxite pollution in the future, which is in accordance with the worldwide mission of transforming “Waste to Wealth”.

## Conflicts of interest

There are no conflicts to declare.

## Supplementary Material

RA-012-D2RA00853J-s001
