# Supplementary material for: Chemoselective decarboxylation of ceiba oil to diesel-range alkanes over a red mud based catalyst under H2-free conditions
Source: RSC Adv. 2022 Jun 8;12(26):16903–17. doi: 10.1039/d2ra00853j (PMC9175285; doi:10.1039/d2ra00853j)
Supplement: RA-012-D2RA00853J-s001 [file RA-012-D2RA00853J-s001.pdf]

Electronic Supplementary Material (ESI) for RSC Advances.
This journal is © The Royal Society of Chemistry 2022

List of supplementary

1. TGA of fresh red mud (RM_x)
2. XRD diffractogram of fresh red mud (RM_x)
3. N_2 adsorption-desorption isotherms of (A) RMO_x, (B) Ni/RMO_x, (C) Ni/rRM$_1$ and Ni/rRM$_3$ and (D) Ni/rRM$_2$ catalysts
4. Pore size distribution (BJH) curves of (A) RMO_x, (B) Ni/RMO_x and (C) Ni/rRM_x catalysts
5. TPD-NH_3 peaks for A) RM_x, B) Ni/RMO_x and C) Ni/RM_x catalysts
6. Alkanes and alkenes n-$(C_{15} + C_{17})$ distribution for RMO_x, Ni/RMO_x and Ni/rRM_x catalysed DO reaction
7. FTIR spectra of deoxygenated liquid product catalysed by (A) RMO_x, (B) Ni/RMO_x and (C) Ni/rRM_x catalysts
8. Gas analysis for Ni/RMO$_3$ catalysed DO. Operating parameter: T = 350 °C, 2 h reaction time, 3 wt.% of catalyst loading.

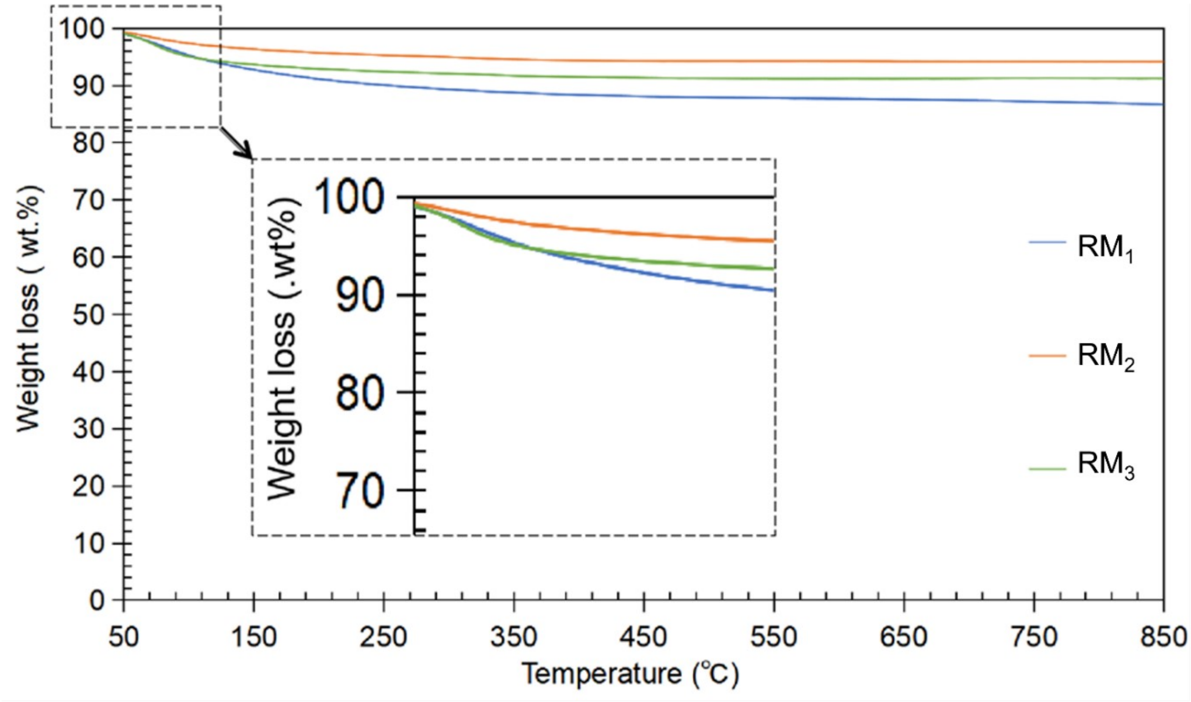

Fig. S1. TGA of fresh red mud (*RM_x*).

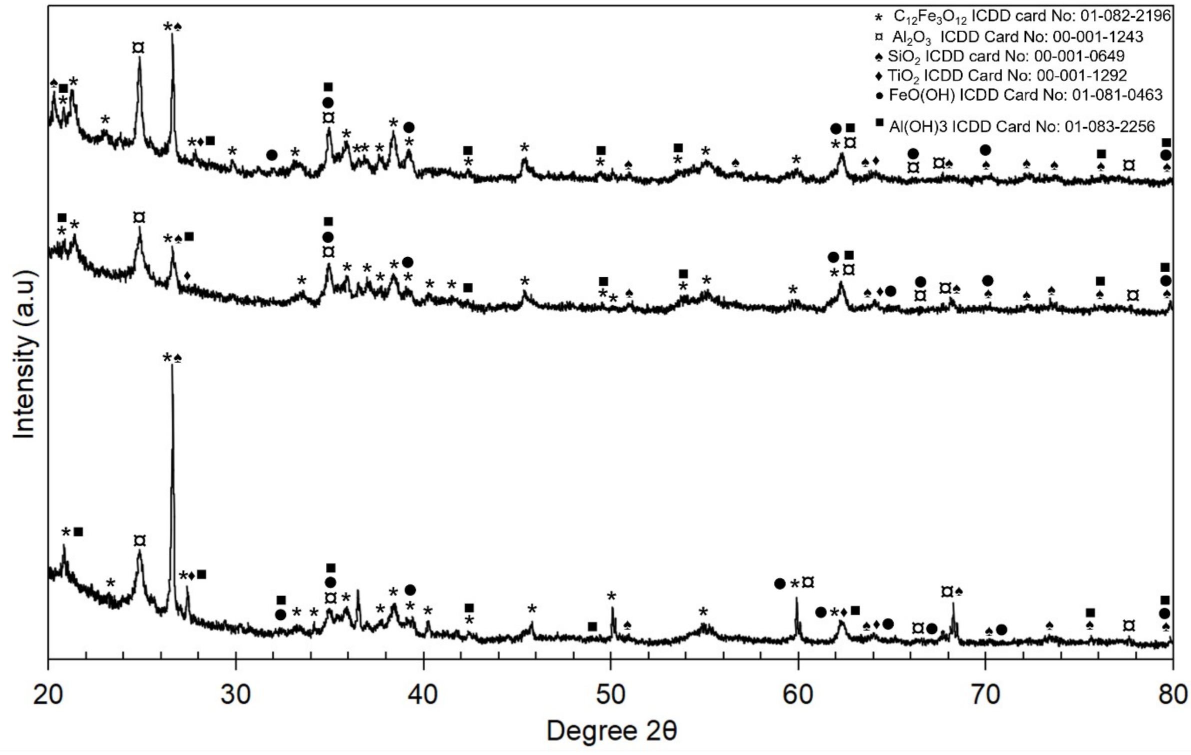

Fig. S2. XRD diffractogram of fresh red mud (*RM_x*).

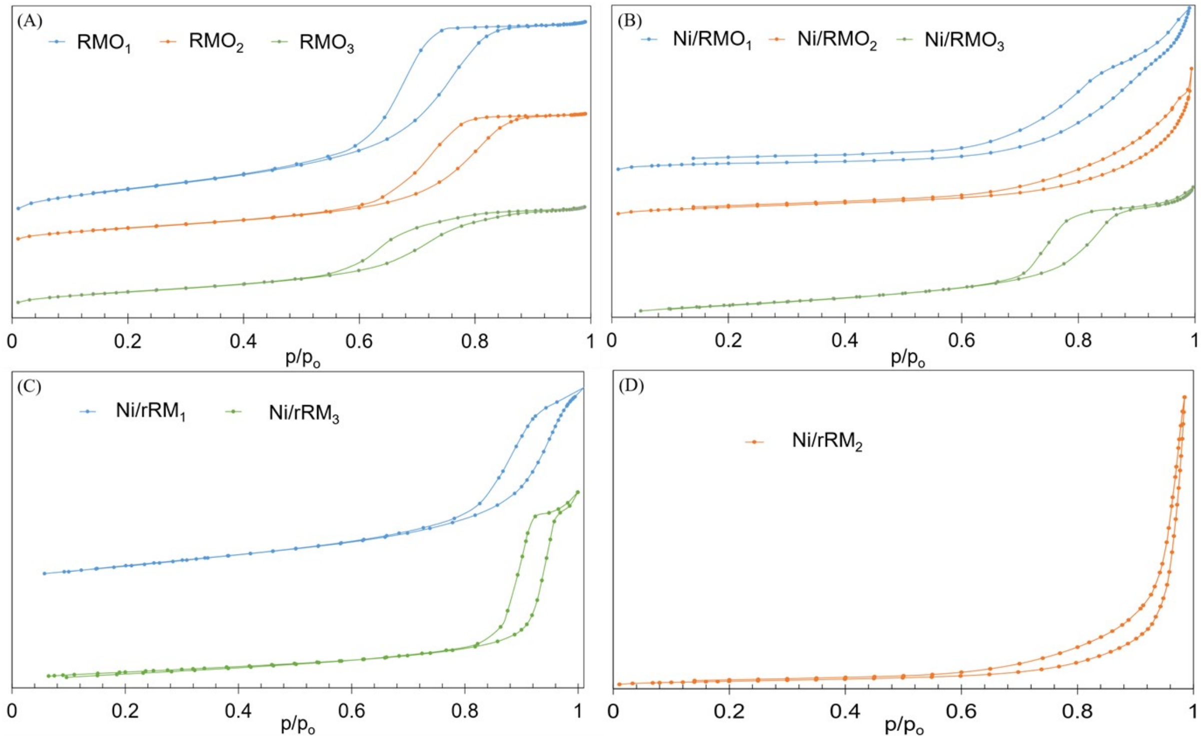

Fig. S3. N_2 adsorption-desorption isotherms of (A) *RMO_x*, (B) *Ni/RMO_x*, (C) Ni/rRM_1 and Ni/rRM_3 and (D) Ni/rRM_2 catalysts.

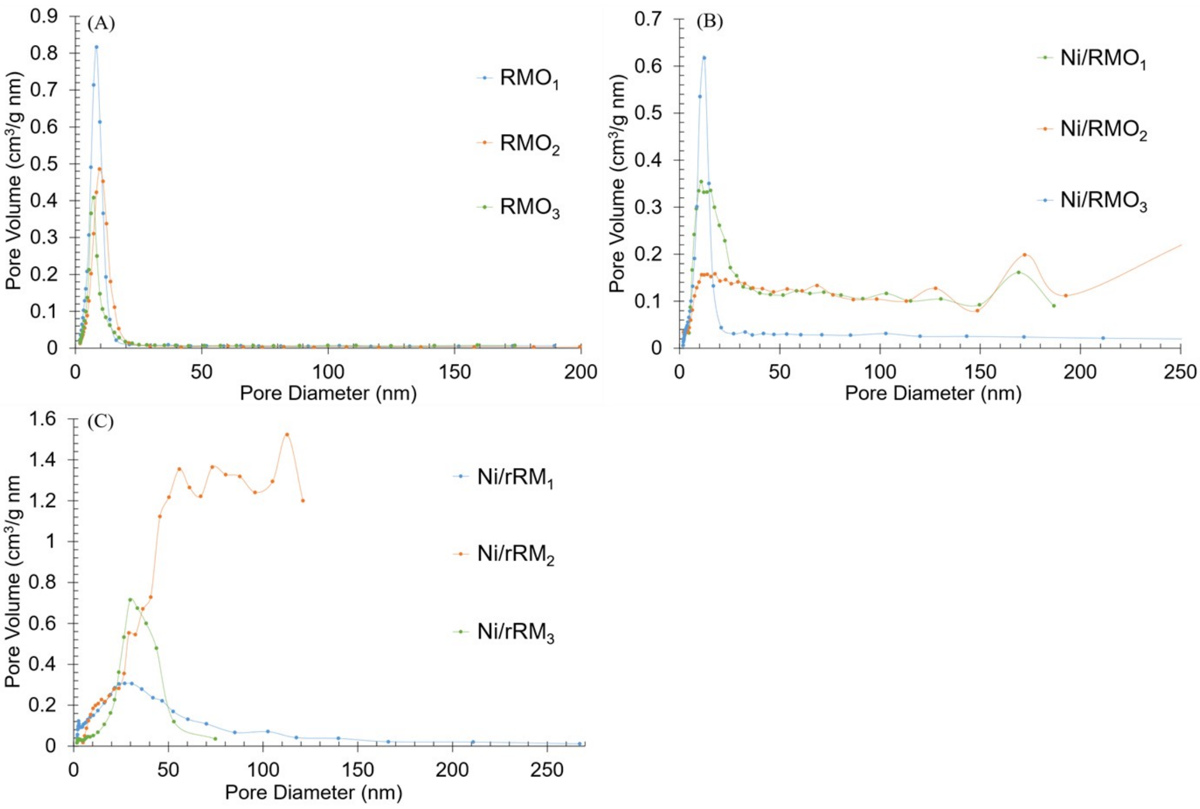

Fig. S4. Pore size distribution (BJH) curves of (A) *RMO*$_x$, (B) *Ni/RMO*$_x$ and (C) *Ni/rRM*$_x$ catalysts.

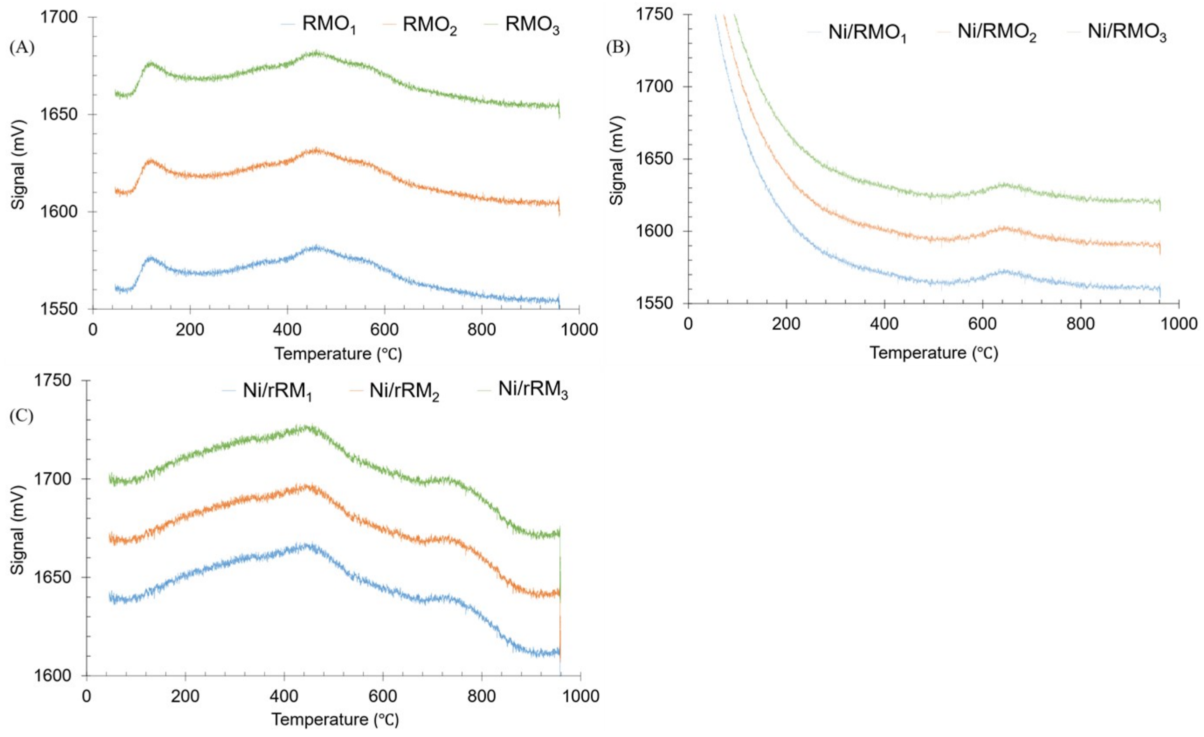

Fig. S5. TPD-NH_3 peak of A) *RMO*$_x$, B) *Ni/RMO*$_x$ and C) *Ni/RM*$_x$ catalysts.

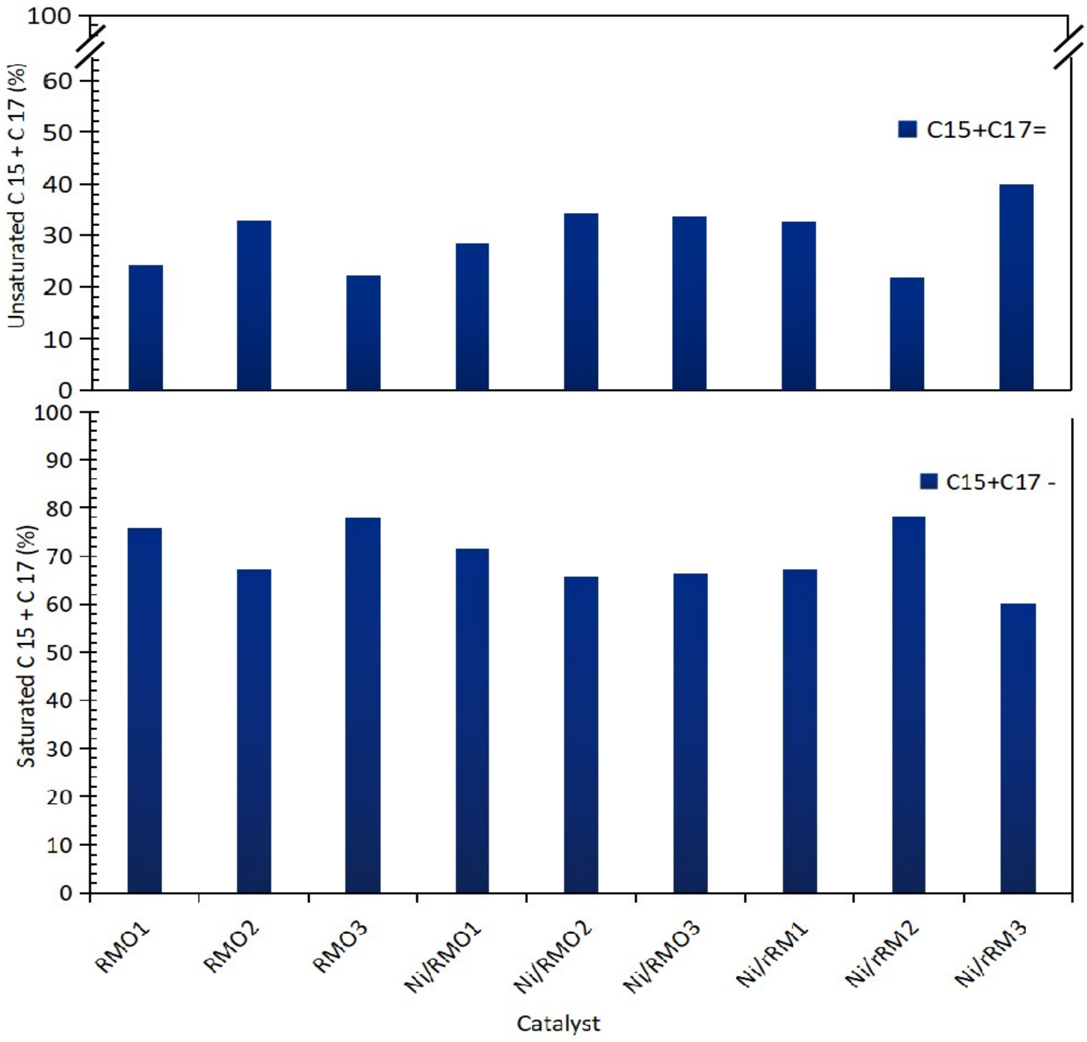

Fig. S6. Alkanes and alkenes *n-*$(C_{15} + C_{17})$ distribution for RMO_x, Ni/RMO_x and Ni/rRM_x catalysed DO reaction. Reaction condition: T = 350 °*C*, 2 h reaction time, 3 wt.% of catalyst loading.

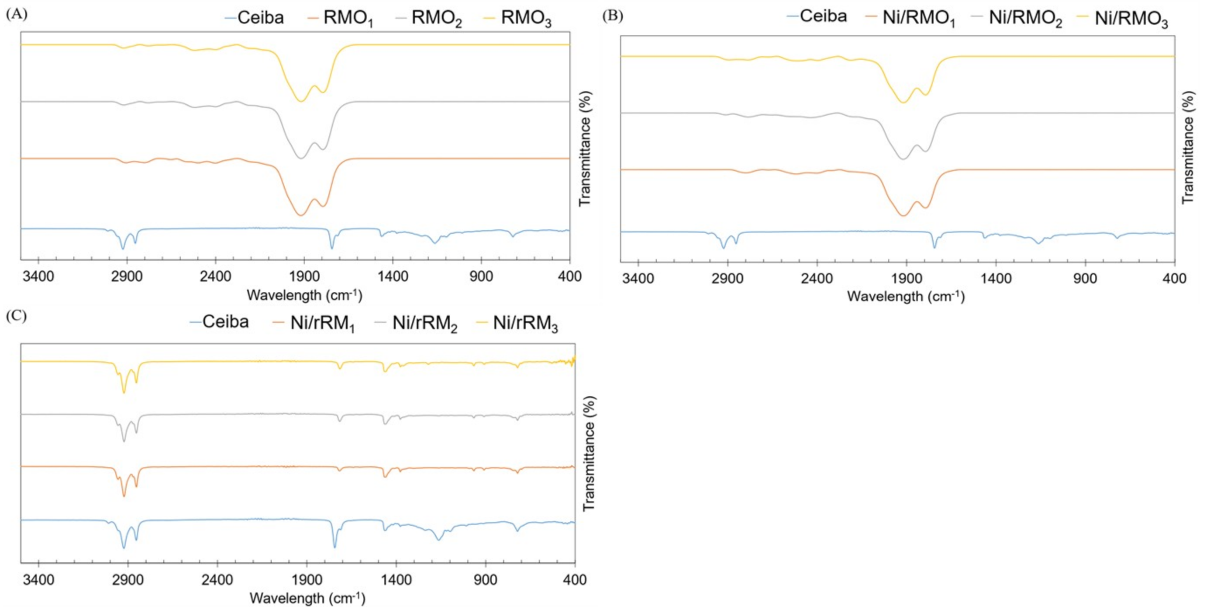

Fig. S7 FTIR spectra of deoxygenated liquid product catalysed by (A) RMO_x, (B) Ni/RMO_x and (C) Ni/rRM_x catalysts

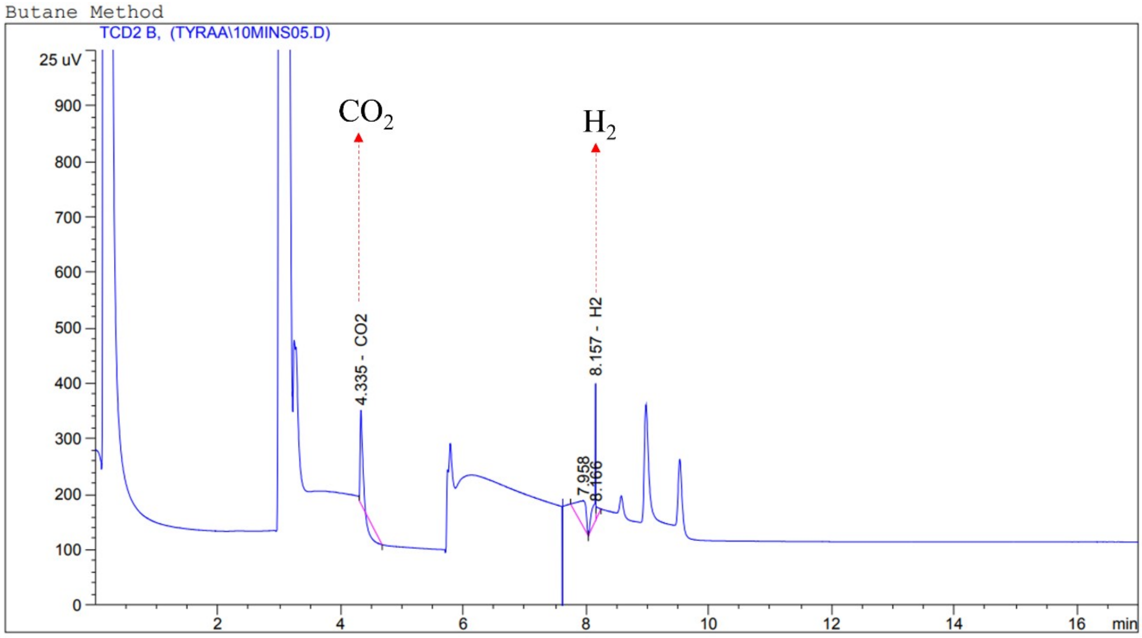

Fig. S8. Gas analysis for Ni/RMO_3 catalysed DO. Operating parameter: T = 350 °*C*, 2 h reaction time, 3 wt.% of catalyst loading.
